# Accessory submaxillary gland: Two new case reports and a literature review

**DOI:** 10.4317/jced.56715

**Published:** 2020-09-01

**Authors:** Jorge Torres-Gaya, Mariano Marqués-Mateo, Delfina Dualde-Beltrán, Álvaro Sada-Malumbres, María del Mar García-San Segundo, Miguel Puche-Torres

**Affiliations:** 1 Oral and Maxillofacial Department. Hospital Clínico Universitario de Valencia; 2 Professor, Surgery Department, Faculty of Medicine and Dentistry, Universitat de Valencia. Valencia, Spain; 3 Department of Radiology, Hospital Clínico Universitario de Valencia; 4 Professor, Department of Medicine, Faculty of Medicine and Dentistry, Universitat de Valencia. Valencia, Spain; 5 Head of Department, Oral and Maxillofacial Department, Hospital Clínico Universitario de Valencia; 6 INCLIVA

## Abstract

**Background:**

The accessory submaxillary gland is a very uncommon anatomical variant, and incidence in the general population has not yet been quantified. The presence of pathology in these glands is rarer still, thus often going unnoticed.

**Material and Methods:**

We describe two accessory submaxillary gland cases, one asymptomatic and the other with chronic sialadenitis in the main and accessory gland caused by sialolithiasis. Although our diagnosis was by computerized tomography, magnetic resonance sialography is helpful to understand and describe this entity with greater precision.

**Results:**

The first case report is an incidental finding and no intervention was required. However, case report number two had clinical symptoms and required a first intervention in which the main submaxillary gland was resected, and a second intervention in which the accessory submaxillary gland was removed. Both patients are asymptomatic to date.

**Conclusions:**

Awareness of the possible presence of accessory submaxillary glands and of potential variations of the excretory ducts is useful in diagnosis, as well as leading to more precise treatment for salivary pathology, and allowing surgeons to avoid complications or injuries during surgery.

** Key words:**Accesory, submaxillary gland, submandibular gland, salivary gland, sialolithiasis, head and neck pathology.

## Introduction

The accessory parotid gland is a well-known anatomical entity with an incidence of 21% in the population (1). However, the accessory submaxillary gland is such a rare condition that its incidence has not yet been documented, and pathology in these glands is even rarer (2). Intriguingly, the accessory submaxillary glands have been widely described and investigated in different bat species (3).

Any salivary tissue (except the major and minor salivary glands) found in the oral cavity, pharynx or upper airway is called heterotopic, most frequently seen in the neck and jaw (4,5). Heterotopic tissue, found in an incorrect anatomical location, is due to embryological aberrations (6). It is important to distinguish it from accessory glandular tissue, regarding which Batsakis (4) postulates that the appearance of these accessory glands is due to the detachment of glandular tissue along the course of the major salivary duct.

To date, including our review, there are only ten duplicative anomalies exclusively reported from the Wharton duct, and nine publications with duplicity of both the duct and the submaxillary gland (eleven cases).

The first reported case of accessory submaxillary gland was published in 1957 by Alexander (7), in a patient with an episode of sialadenitis diagnosed by conventional sialography. A publication dating from 1950 under the name of Figun (8) could in fact be the first accessory submaxillary gland described, but the text was inaccessible. Codjambopoulo *et al.* (9) published the first case describing bilateral duplication of both the submaxillary gland and the duct. Gadodia *et al*. (10) published the first case of sialadenitis, due probably to sialolithiasis (the referred patient had expelled a small calculus prior to the imaging tests), in the accessory submandibular gland identified by magnetic resonance (MR) sialography. Bryan *et al.* (2) published the first documented case of a pleomorphic adenoma within an accessory submaxillary gland in 2013. It was identified by ultrasonography, diagnosed by Fine Needle Puncture Aspiration (FNA) cytology and finally during surgery the presence of an accessory gland with a tumor completely separate from the main submaxillary gland was verified.

The most recently documented pathological case was published by Sánchez Barrueco *et al.* (11). The patient was clinically diagnosed with sialolithiasis, and subsequently underwent computarized tomography (CT) and MR sialography, confirming the calculus in an accessory submaxillary gland.

As an anatomical and clinical principle, any existing pathology of the main salivary gland can appear in an accessory salivary gland (12), although these duplicative anomalies are mostly asymptomatic, so it seems reasonable that only those associated with pathology of the gland or the duct would require treatment (10).

The objective of the present manuscript is to report two new cases of accessory submaxillary gland, one as a casual finding during cancer staging in a patient with oral oncological pathology, and another in a patient with symptoms of sialadenitis and sialolithiasis in accessory and principal gland.

## Case Report

-Case 1

**Figure 1:**
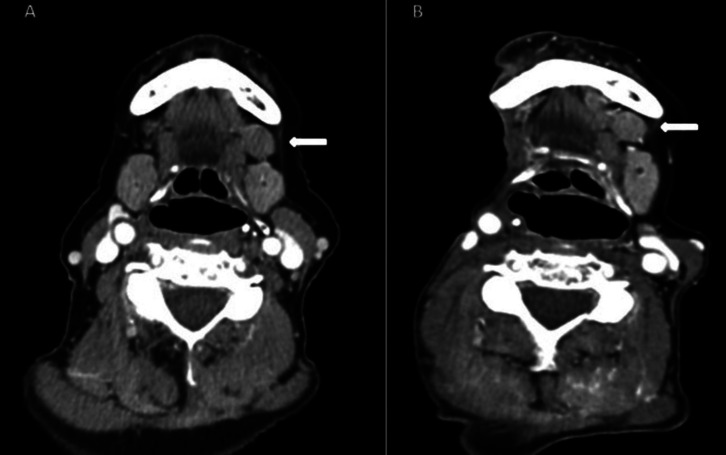
A) Axial cervical CT after administration of intravenous contrast (iv) in which a left accessory submaxillary gland is observed in front of the main gland. B) Cervical CT with same image on the left side two years after the right cervical emptying.

A 75-year-old woman presented with oral carcinoma, and during radiological examinations performed in 2014 to assess cancer stage, a suspected double submaxillary gland was found in CT scan. Contralateral adenopathy was considered in the differential diagnosis. Fine Needle Puncture Aspiration (FNA) confirmed the presence of glandular tissue. At two-year follow-up after the oncological intervention CT showed the same image without changes (Fig. 1). The patient had no glandular symptoms on the left side at any time.

-Case 2

**Figure 2:**
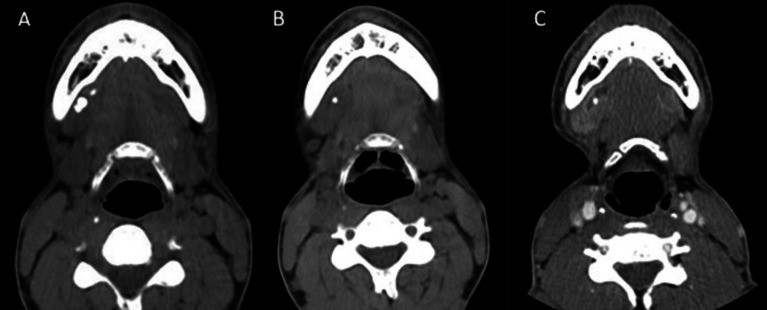
A) Initial axial cervical non-enhanced CT scan with right main and accessory submaxillary glands and lithiasis in the Wharton’s duct. B) Axial cervical non-enhanced CT scan with accessory submaxillary gland and reparative scar tissue after the first intervention. C) Axial cervical CT scan on administration of iv contrast after the second intervention, with absence of the right submaxillary glands, dilation of the right Wharton’s duct and calculus in its interior.

After multiple episodes of right submaxillitis a 37-year-old man underwent a CT scan in 2012, in which two calculi were seen in the right Wharton duct. A new episode of submaxillitis required hospitalization, and during this stay they were expelled. Subsequently, surgery was performed for right submaxillectomy, and histopathological study confirmed chronic atrophic sialadenitis in a 3.2x2cm piece. After one year the patient presented again with right submaxillitis and on CT a formation compatible with a remnant submaxillary gland was observed. A new intervention confirmed the existence of a gland with 3x2cm chronic inflammation. In later checkups a dilated Wharton duct was observed, with a 3 mm calculus in its interior over an enlarged sublingual gland (Fig. 2). The patient remains asymptomatic two years after the last intervention.

## Discussion

The submaxillary gland is one of three major salivary gland pairs. It can present with obstructive pathology such as sialolithiasis, inflammatory pathology such as sialadenitis and benign or malignant tumor pathology. In contrast to presence of the accessory parotid gland, the duplicity of the gland or accessory ducts of the submaxillary gland are principally testimonial reports and incidence in the population has not been quantified.

The submaxillary accessory duct is a rare entity and a literature search yields limited published works. Even more exceptional is the presence of accessory submaxillary glands. Only nine papers appear in the literature (2,6,7,9-14). Table 1 describes the published cases. In both entities, low prevalence results in few searches, which contributes to their relative obscurity. Of the published works, some refer to findings during anatomical dissection (14,15), others to the presence of pathology in those areas: obstructive alterations such as sialolithiasis (11,16,17) or even tumors, for example the presence of a mixed tumor (2,6).

**Table 1:**
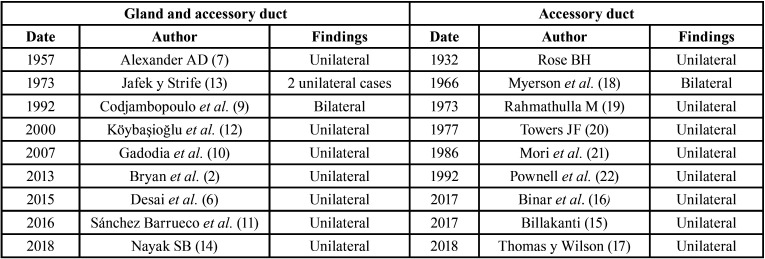
Published cases of accessory glands and accessory ducts to date.

Knowledge regarding duplicity of independent exit ducts or the presence of accessory glands can therefore prove essential for radiologists and head and neck surgeons.

Conventional preoperative practices in submaxillary gland surgery do not include investigation for possible anomalies in this area. In our protocol, we initially request a CT scan to assess cervical soft tissue pathology. In the first case presented, the CT was to evaluate cervical oncological pathology, and in the second to assess the presence of cellulitis in the submandibular space. Subsequently, we were able to confirm the presence of accessory submaxillary glands. In the first case this was a casual finding during follow-up of an oncological patient, and in this case was of purely anatomical interest. This highlights that when there are no clinical findings or when the diagnosis of accessory tissue is not taken into account, the surgeon may encounter anatomical variations and possible intraoperative surprises. In the second case, after the appearance of a new episode of sialadenitis in the same location we should have suspected the presence of this anomaly, which was confirmed once the submaxillary gland was removed for the second time and the CT images prior the first intervention were reviewed (Fig. 2).

MR Sialography is currently accepted as the best method for studying the salivary glands (11,22) and can be used to detect accessory glands and ducts, but is not a standard first imaging procedure owing to its cost. However, as a non-invasive imaging test, without the need for ionizing radiation, and with a high capacity for tissue discrimination, which allows it to identify in detail not only the glandular tissue but also the ductal system of the submaxillary gland (11), it seems an optimal procedure to rule out this anatomical variant.

## Conclusions

Although particularly rare, pathologies affecting the accessory submaxillary glands must be considered in the differential diagnosis of submandibular area lesions. Surgeons should check for presence of anatomical variations of the submaxillary gland or duct to avoid complications. If these variations are suspected, MR Sialography is justified prior to intervention.
